# A policy analysis of active travel in public healthcare organizations, Victoria Australia

**DOI:** 10.1093/heapro/daaf192

**Published:** 2025-11-25

**Authors:** Richard Larsen, Fiona Dangerfield, Glenda Verrinder

**Affiliations:** La Trobe University, La Trobe Rural Health School, PO Box 199, Bendigo, Victoria 3552, Australia; La Trobe University, Violet Vines Marshman Centre for Rural Health Research, PO Box 199, Bendigo, Victoria 3552, Australia; La Trobe University, La Trobe Rural Health School, PO Box 199, Bendigo, Victoria 3552, Australia

**Keywords:** active travel, organizational policy, healthcare, environmental sustainability

## Abstract

Healthcare organizations contribute directly and indirectly to global greenhouse gas emissions and consequently to degradation of the environment and poor human health. Active travel has known positive effects on both the health of the environment and humans. Within Australia, healthcare organizations are significant employers and while efforts to reduce the impact of healthcare organizations on the environment are growing, there remains little advancement in increasing rates of active travel among healthcare staff. This study describes active travel policies in the public healthcare sector within the State of Victoria in Australia through a systematic analysis of publicly facing documents. A qualitative document analysis approach was adopted to investigate Victorian public healthcare organization’s policies for active travel. Organizational websites, environmental policies, health promotion pages, and vision and mission statements were analysed for active travel policies and content. Of the 21 healthcare organizations investigated, none had a specific policy on active travel for its employees. Many organizations had environmental sustainability policies, with some policies referencing the need to transition staff into more sustainable commute modes. The inclusion of measurable policy objectives was mostly absent. There is a paucity of policies that would enable an increase in active travel in the major publicly funded Victorian healthcare organizations in Australia, despite the majority of organizations signing on to the Global Green and Healthy Hospitals Initiative, which highlights patient and staff transport as one of the ten sustainability goals. Without clear and actionable policy, healthcare organizations will remain at a difficult crossroads where their culture fails to reflect the health promoting advice they provide to their community. However, with this knowledge, opportunities such as improving end of trip facilities and the development of robust facilitative policy can be undertaken.

Contribution to health promotion:

- There have been advances in sustainability policy within the healthcare sector; yet, there remains a lack of policy regarding employee commuting to reduce scope 3 emissions.
- Few healthcare organizations in Victoria, Australia set targets towards reducing motorised commuting and increasing active commuting.
- Future sustainability policies within healthcare organizations should consider staff commuting to facilitate the transition towards a healthier and more environmentally sustainable sector.

## INTRODUCTION

Healthcare contributes ∼5% of global emissions (Pichler et al. 2019), a figure expected to rise with increased demand on the healthcare sector anticipated in the decades to come (Or and Seppänen 2024). In Australia, this figure is similar, with ∼5.5% of emissions stemming from healthcare, the majority of which (∼59%) are from healthcare services such as hospitals, as opposed to residential care (33%) and pharmaceutical and product manufacturing (8%) (De Sain 2024). This produces a major challenge for these organizations within the context of environmental sustainability and public health. The greenhouse gas emissions produced by the healthcare sector are a contributor to climate-related health risks from heatwaves, fires, floods, and diseases (Zhang et al. 2018, Beggs et al. 2021, Romanello et al. 2021, Lee et al. 2023, Turner et al. 2023). Without interventions to reduce healthcare related greenhouse gas emissions, healthcare organizations will continue to sit at the uncomfortable junction between contributing indirectly to climate health risks, while trying to rectify that harm through the care they provide. Healthcare organizations can play a significant role in reducing the direct and indirect health risks caused by rapidly rising greenhouse gas emissions and work towards improving population health in a changing climate (Malik et al. 2018, Zhang et al. 2018, Beggs et al. 2021, Romanello et al. 2021, Lee et al. 2023, Or and Seppänen 2024).

The awareness of the negative impact healthcare organizations are having on the environment has been steadily increasing, with ‘green healthcare’ a prominent issue within healthcare organizations globally. Initiatives such as Global Green and Healthy Hospitals, Health Care Without Harm, and Practice Greenhealth have all been established in response to the recognition of healthcare’s contribution to climate change. As a result, the development of policies to reduce greenhouse gas emissions within the healthcare sector has increased (Malik et al. 2018) and Australia’s health system is working towards a net zero healthcare system, with all states and territories to become net zero by 2050 (Verlis et al. 2024).

Policies to reduce greenhouse gas emissions have typically focused on Scope 1 and Scope 2 emissions (Thanekar et al. 2024). Scope 1 emissions are direct greenhouse gas emissions from sources owned or controlled by an organization (e.g. electricity generation, emissions from fleet vehicles), while Scope 2 emissions are referred to as indirect emissions (emissions from purchased energy) (Thanekar et al. 2024). There remains limited information on reducing emissions from indirect sources or Scope 3 emissions, for example, transport and disposal of waste; and commuting to and from work (Thanekar et al. 2024).

Commuting is a significant scope 3 emission across the world, not just within the healthcare sector. Nations such as Australia lead the world in car commuting, with over 50% of commutes undertaken by motorised vehicles (Australian Bureau of Statistics, 2025). This figure is expected to increase as Australia moves out of the COVID-19 pandemic and commuting becomes more frequent again. Furthermore, healthcare organizations are significant employers across both metropolitan and regional areas in Australia, with ∼5% of the total population employed in the sector (Australian Institute for Health and Welfare, 2024). This therefore makes the healthcare sector’s commuting practices a significant source of greenhouse gas emissions.

A range of solutions are available to reduce the negative consequences of employee commuting within an organization. These include increasing the use of public transportation, carpooling, and working from home policies (Burtnik Urueta et al. 2025). This research investigates the potential of active travel in reducing employee commuting-related emissions. Active travel, the use of a physically active transportation mode such as cycling or walking, both reduces the environmental cost and the human health consequences of commuting when compared to motorised travel (Caldwell and Boyer 2018, Beggs et al. 2021, Brand et al. 2021, Gardner et al. 2023, Beck et al. 2022). Despite the knowledge that active travel provides a solution to a multi-faceted problem, active travel rates remain low in Australia, with just 5% of trips taken by bicycle or walking (Beck 2021). This is lower than comparable western nations such as the Unites States of America (12%) (Ghimire and Bardaka 2023) and England (33%) (Jessiman et al. 2023, UK Department of Transport, 2021).

Facilitative policy can play a role in augmenting the transition from motorised to active travel (Friman et al. 2013, Yang et al. 2015, Pearson et al. 2023, Hunter et al. 2025). Yet, research and policy are often at a disconnect (Nutbeam 2010, Buse et al. 2012, Giles-Corti et al. 2015, Bogenschneider 2021), with ‘tensions between those who seek to understand the world and those who seek to govern it’ (page 36 (Bogenschneider 2021)) a commonly reported barrier. In part, this may be attributed to the gap between research scholars and policy makers, whereby research scholars rarely obtain exposure to the world of policymakers and vice versa (Giles-Corti et al. 2015).

It is unknown what emphasis major Victorian healthcare organizations place on employee active travel and if they have developed policies to assist their workforce transition away from motorised commuting. Organizational change within the realm of climate change and sustainability is often slow, misguided, and/or inadequate to produce meaningful contributions to climate change mitigation (Finke et al. 2016, Slawinski et al. 2017). Short termism and uncertainty are cited as common barriers (Slawinski et al. 2017), which can arise from economic concerns, for example, the up-front costs of building end of trip facilities, and from an embedded culture that fails to adapt to a changing world. Both concepts can be overcome through robust top-down leadership and the development of progressive policy (Finke et al. 2016, Petrunoff et al. 2017, Slawinski et al. 2017). Therefore, the aim of this study was to analyse Victorian public healthcare policies to describe active travel and identify opportunities to translate policy into action. In doing so, this research asks: How is active travel currently framed within healthcare organization’s policy? What gaps exist within healthcare organizations regarding active travel policy? And what opportunities are there for strengthening policy to better support employee active travel?

## METHOD

### Study design

Taking a pragmatist approach to qualitative document analysis, we examined policy documents while drawing on established policy analysis frameworks (Bowen 2009, Taylor et al. 2024). Qualitative document analysis was undertaken, as this study design provided a structured and systematic method for analysing the content of healthcare organization’s policy documents and is a commonly used approach in health policy analysis (Kayesa and Shung-King 2021). Rigour was maintained by following guidelines set out by Kayesa and Shung-King 2021, whereby clear processes for searching and identifying policy documents was developed along with a codebook for search terms. Content extracted from the policy documents were then recorded for analysis alongside the codebook. A pragmatist approach was adopted to guide the qualitative document analysis, as this was suited to policy research due to its prioritization of practical inquiry and the generation of actionable knowledge, rather than adherence to a single theoretical lens (Morgan 2014). This approach focused the central aim of understanding how active travel is represented within healthcare policy and what opportunities exist for translating policy into practice.

The positionality of the researchers was also acknowledged. The lead researcher (R.L.) works within the public health sector and recognised that this professional background could shape perspectives brought to the analysis, even while using a previously reported analysis structure (Bowen 2009). While this familiarity risked introducing assumptions, it also provided valuable contextual insight into the policy environment. Ongoing reflection was undertaken through meetings with the coordinating researcher (G.V.), who is not employed within the sector, to ensure that these dual influences were not impeding collection or analysis.

### Study setting

Australia’s health system is a complex mix of service providers across a range of organizations. These include federal, state, and territory governments, and private sector service providers. Victoria has over 300 hospitals and other health services, including large public and private hospitals, rural and regional health services, community health centres, women’s health services, specialist mother and child services and small specialist rehabilitation and psychiatric hospitals. Health services are grouped via geographical region into Local Health Service Networks. Within the state of Victoria, both metropolitan and regional areas have six local networks that provide care to their designated area. The provision of local care networks aims to ensure care is provided as close to home as possible. This study included all Victorian metropolitan publicly funded healthcare organizations (*n* = 14) and a purposive sample of major Victorian regional healthcare organizations (*n* = 7) (Table 1). All rural and bush nursing healthcare organizations were excluded due to the variability of staff travel distances, which may impact on levels of active travel (Pearson et al. 2023).

**Table 1.** Purposive sample of metropolitan and major regional health services in Victoria that were included for policy analysis.

Health service name	Classification	Health service network
Alfred Health	Metropolitan	Bayside
Austin Health	Metropolitan	North Metro and Mitchell
Calvary Bethlehem Health	Metropolitan	Bayside
Eastern Health	Metropolitan	East Metro and Murrindindi
Melbourne Health	Metropolitan	Parkville
Mercy Health	Metropolitan	West metro
Monash Health	Metropolitan	South metro
Northern Health	Metropolitan	North Metro and Mitchell
Peninsula Health	Metropolitan	Bayside
Peter MacCallum Cancer Centre	Metropolitan	Parkville
St Vincent’s Health	Metropolitan	East Metro and Murrindindi
Royal Children’s Hospital	Metropolitan	Parkville
Royal Women’s Hospital	Metropolitan	Parkville
Western Health	Metropolitan	West metro
Albury Wodonga Health	Regional	Hume
Bairnsdale Regional Health	Regional	Gippsland
Barwon Health	Regional	Barwon
Bendigo Health	Regional	Loddon Mallee
Goulburn Valley Health	Regional	Hume
Grampians Health	Regional	Grampians
South West Healthcare	Regional	South west

Given all data collected and analysed were publicly available, no ethical approvals were sought for this work.

### Data collection

Data collection was undertaken in 5 steps.

#### Step 1: search of organization’s website for policy documents

A search of 21 Victorian public healthcare organization websites was conducted by (R.L.) between March and April 2025 to identify and download publicly available policy documents that may contain active travel related policies. The search terms used were informed by results from a systematic review of the literature (Larsen et al. 2024) and included the following: ‘active travel; active transport; cycling; bicycle; walking; end of trip facilities’. Areas of websites and policy documents that contained a keyword search hit were examined further to identify if the healthcare organization had one or more of the following: an active travel policy; information on end of trip facilities; an environmental sustainability policy.

#### Step 2: google search

A Google search was subsequently performed to identify any content not discovered during the search for policy documents. The following search terms were used for each healthcare organization: ‘[health service name] environmental sustainability; [health service name] bicycle; [health service name] global green and healthy hospitals’.

#### Step 3: review of policy documents

A keyword search of the identified policy documents was conducted to determine the relevance of the document to active travel. The search terms used were the following: ‘Active travel, active transport, cycling, bicycle, walking, end of trip facilities’. Only policies that were current were included in the study.

#### Step 4: review of health service’s health promotion content

Each organization’s publicly facing health promotion section of their website was specifically searched using the following terms to identify if there was a focus on active travel: ‘Active travel, active transport, cycling, bicycle, walking, end of trip facilities’.

#### Step 5: review of organizational vision and mission statements

Each health organization’s website was searched for their current vision and mission statements to identify if there was a focus on environmental sustainability.

## DATA ANALYSIS

Environmental sustainability policy documents and organization’s health promotion websites were analysed using conceptual content analysis (Bengtsson 2016) to identify the following key search terms: ‘active travel, active transport, transport, cycling, bicycle, walking, end of trip’ facilities. Phrases that matched the key search terms were entered into an Excel spreadsheet and coded according to their search term. Following this initial coding, we examined the surrounding text of each reference to understand how the terms were used within the policy context. Extracted segments were then compared across documents and grouped according to recurring ideas or positions. This process enabled the development of themes that reflected how active travel was prioritized, the types of strategies proposed, and the extent to which implementation considerations were addressed.

The above content analysis in combination with the Google search of organization’s websites provided a breakdown of end of trip facilities at each organization and whether or not the organization was a member of Global Green and Healthy Hospitals. End of trip facilities were categorised into three separate amenities: ‘bicycle parking, lockers, showers’. Each organization was classified according to the presence or absence of these facilities. Similarly, each organization was classified as being a member of Global Green and Healthy Hospitals or not.

Vision and mission statements were extracted from organization’s websites into a spreadsheet for analysis. A focus on environmental sustainability was assessed by reviewing the text for the following words: ‘sustainability, sustainably, environment, environmental’.

## RESULTS

Our analyses identified that no Victorian public healthcare organization included in this study had a specific active travel policy for its employees. Many organizations had a form of environmental or sustainability policy, and some of these documents referenced the need to transition staff into more sustainable commute modes.

### Environmental sustainability policies

Across the 21 healthcare organizations, 57% (*n* = 12) had a current environmental sustainability policy (Table 2). Of the 14 metropolitan healthcare organizations, 64% (*n* = 9) had current environmental sustainability policies. Of the 7 regional healthcare organizations, 57% (*n* = 3) had current environmental sustainability policies. Despite 71% (*n* = 15) of healthcare organizations being a member of GGHH, only 57% (*n* = 11) had a current environmental sustainability policy (Table 2). Of the 12 current environmental sustainability policies identified, 58% (*n* = 7) included statements referring to active travel. Examples of specific goals referring to active travel are presented below.

Promote and encourage staff, patients and visitors to choose environmentally sustainable transport options

- Alfred Health, Environmental Sustainability Strategy 2022–2025.
  Develop new transport initiatives for staff travelling to work, for example car share systems, systems that favour public transport, or active transport (walking/bike)
- Mercy Public Hospitals, Caring for People and Planet 2022–2025.
  Encourage active travel (e.g. walking, cycling) and sustainable modes of transport for our staff and community
- Monash Health, Environmental Sustainability Policy 2024.
  10% increase from current levels in staff using sustainable transport such as public transport, cycling, walking, carpooling, electric/hybrid vehicles, shuttle services, or working remotely
- Peter McCallum Cancer Centre, Environmental Sustainability Strategy.
  Promote and transition to sustainable transportation and reduce the need to travel
- St Vincent’s Health, Environmental Sustainability Action Plan 2024–2027.
  Implement programs that expand the use of sustainable forms of transport such as walking, cycling, public transport, carpooling and using fuel efficient vehicles for work journeys
- Royal Women’s Hospital, Environmental Management Plan 2022–2027.

Regional health organizations also included active travel and sustainable transport in their policies but to a lesser extent than metropolitan healthcare organizations. Examples included:

Collaborate with relevant stakeholders to improve access to public transport

- Barwon Health, Environmental Sustainability Strategy 2024–2030.

We note that the majority of the above statements were unspecific and immeasurable and did not provide an action point or mechanism of monitoring for improvement. All but one policy provided process terms such as, ‘encourage’, ‘promote’, ‘improve’ or ‘investigate’. One policy included a goal-orientated statement indicating an aim for a ‘10% increase from current levels in staff using sustainable transport such as public transport, cycling, walking, carpooling, electric/hybrid vehicles, shuttle services, or working remotely’ (Peter McCallum Cancer Centre). This was the only example in the reviewed policy documents providing a measurable approach to increasing active travel for their staff.

**Table 2.** Overview of victorian healthcare organizations (*n* = 21) active travel and environmental sustainability policies.

Health service name	Active travel policy	Environmental sustainability policy	End of trip facilities	Member of GGHH
Alfred Health	No	Yes	Storage, lockers, showers	Yes
Austin Health	No	To be published in June 2025	Bicycle parking	Yes
Calvary Bethlehem Melbourne	No	Yes	No	No
Eastern Health	No	No	Bicycle parking	Yes
Melbourne Health	No	Yes	Bicycle parking	Yes
Mercy Public Hospitals	No	Yes	Bicycle parking	No
Monash Health	No	Yes	Bicycle parking	Yes
Northern Health	No	Yes	Bicycle parking	Yes
Peninsula Health	No	No	Bicycle parking (paid)	Yes
Peter MacCallum Cancer Centre	No	Yes	Storage, lockers, showers	Yes
St Vincent’s Health	No	Yes	Bicycle parking	No
Royal Children's Hospital	No	No	Storage, lockers, showers	Yes
Royal Women's Hospital	No	Yes	Storage, lockers, showers	No
Western Health	No	Not current	Storage, lockers, showers	Yes
Albury Wodonga Health	No	No	No	No
Bairnsdale Regional Health	No	No	Storage, lockers, showers	Yes
Barwon Health	No	Yes	Bicycle parking	Yes
Bendigo Health	No	Yes	Bicycle parking	Yes
Goulburn Valley Health	No	No	No	Yes
Grampians Health	No	Yes	No	No
South West Healthcare	No	Not current	No	Yes

### Health promotion and active travel website content

When reviewing each healthcare organization’s health promotion webpages, 2 of the 14 metropolitan and 1 of the 7 regional healthcare organizations included a focus on active travel. One organization stood out as an exemplar, with a specific focus on improving bicycle end of trip facilities following consultation with an external professional body.

We encourage staff and visitors to use active forms of travel to and from our sites. We've recently upgraded facilities to support active travel to Alfred Health. We worked with Bicycle Network to review how staff get to work, and investigate requirements to further encourage active travel. In response to feedback from staff, we built the Active Travel Zone (ATZ): a secure staff facility equipped with 300 bike parks, 237 lockers, showers, change rooms and secure electronic access. (Alfred Health, Healthy Communities webpage).

One metropolitan healthcare organization briefly included National Walk Safely to School Day as an initiative they supported. One regional healthcare organization briefly included the benefits of active travel and indicated they supported its uptake to improve the health of the community.

No organizations included the importance of reducing carbon emissions as a means of improving health. Similar to the statements included in environmental sustainability policies, health promotion webpages did not include specific statements on increasing active travel, for example, ‘Increasing active living’, ‘working with the community to increase opportunities for physical activity’ and ‘provide an environment that promotes healthy and active living for our community’. Much of the health promotion content within the organization’s websites was broad and did not provide prescriptive advice to individuals on how to improve their health e.g. ‘All too often, our busy lifestyles get in the way and stop us from being as active as we should’.

### End of trip facilities

The majority (13 of 14) of metropolitan services had some form of end of trip facilities (one or a combination of bicycle parking, lockers and/or showering facilities). However, of the 14 organizations, only 5 indicated they had a purpose-built combination of bicycle parking, lockers and showering facilities available to staff. Just three out of the seven major regional healthcare organizations indicated they had end of trip facilities, with just one organization having all three of bicycle parking, lockers, and showers. An overview of metropolitan and major regional healthcare organizations active travel policy and end of trip facilities is presented in Table 2.

### Vision and mission statements

Vision and mission statements included phrases such as ‘our promise’, ‘strategic priorities’, ‘strategic goals’, and ‘values’. Only 2 of the 21 health services explicitly highlighted sustainability in their phrases. For example:

We manage our resources wisely and sustainably to provide value for our community (Monash Health)

Sustainability, Systems and Infrastructure—We will design and facilitate the delivery of progressive and sustainable healthcare (Peninsula Health)

## DISCUSSION

The aim of this study was to describe active travel policy within Victorian public healthcare organizations. This process was undertaken to identify what policies, if any, currently exist, and to find opportunities to translate policy into action.

### Environmental sustainability policy

Despite our findings indicating there are no specific active travel policies publicly available, many Victorian healthcare organizations did have environmental sustainability policies in place. The focus of these policies was largely on renewable energy production and waste reduction. Renewable energy production is an obvious focus given global transition away from coal and gas, as well as the benefits this has for long term energy provision (Beggs et al. 2021). Waste reduction is also front of mind in hospitals, as single use items continue to dominate (Thanekar et al. 2024). The waste generated from single use items is a significant economic overhead to dispose of, while also contributing to landfill and pollution (Braschi et al. 2022).

Notably, the policy documents that did include statements on active travel were rarely specific or measurable in their outcomes. Considering the results, we argue the lack of focus and inaction will continue to place healthcare at odds with their public health advice and care they provide. Previous research within the healthcare sector has identified staff are eager to transition away from motorised vehicle commuting if they have the support from their organization (Larsen et al. 2025). However, there remains a lack of managerial leadership to provide adequate policy that will lead to meaningful results (Petrunoff et al. 2017, Walker et al. 2022, Goddard 2024, Pearson et al. 2024, Larsen et al. 2025, Pearson et al. 2025). Parking management is a useful example when considering the role of leadership in active travel and is presented insightfully by Petrunoff and colleagues who argue the loss of parking availability can be seen as an enabler, rather than a barrier, to policy development that promotes and increases active travel. Organizations require ‘top-down’ leadership to provide built-for-purpose end-of-trip facilities that make active commuting an inviting and feasible option (Larsen et al. 2025). We suggest policy documents cannot be merely placeholders that provide the image of environmentalism. Rather, they need to be actionable and informative to the staff operating within them.

It is worth highlighting that policy development is a dynamic process with many influences and actors and not simply the production of a statement. Furthermore, research often fails to translate adequately into policy, a phenomenon well documented elsewhere (Nutbeam 2010, Buse et al. 2012, Kelly and Barker 2016, Gentry et al. 2020, Kilbourne et al. 2022). This can be for a multitude of reasons that are often specific to the context and research domain. In this case, we observe a lack of organizational focus on reducing motorised travel as a significant contributor. All but one organization showed a lack of direct pursuit to increase active travel through promotion and investment in infrastructure. Prior research indicates the importance of organizational culture in improving active travel rates, a process that can take time (Aldred and Jungnickel 2014). It is therefore imperative for policy to incorporate research findings from behavioural science and urban design to instigate a cultural shift. Australia is a car-centric nation where motornormativity dictates travel plans (Walker et al. 2022, Goddard 2024). Hence, fostering change towards sustainable modalities of travel such as cycling and walking is more difficult and faces greater resistance than in cultures where these modes are accepted (Gardner et al. 2023).

These challenges are, in part, contributed to by the political and social environments of nations and organizations (Legacy 2015, Gentry et al. 2020). Political agendas often dictate the direction of public health pursuits. We observed a significant focus on the transition to renewable energy and reduction in waste generated from hospitals within the included policies. This is a worthwhile focus but may come at the expense of other areas of carbon reduction (Buse et al. 2012, Giles-Corti et al. 2015, Gentry et al. 2020). The complexity of the political environment also plays a role, with differing levels of government (local, state, and federal) overlapping in their responsibilities. This can create confusion and a transfer of responsibility. Active travel is also an inherently controversial topic, creating binary viewpoints that may lead to dispute and inaction (Legacy 2015, Slawinski et al. 2017, Verlinghieri et al. 2024). Leadership and policy development is therefore imperative to prevent inaction and short termism that will hinder the advancement of active travel.

### Health promotion and active travel

Planetary health and human health are inherently linked, and health promoting policy must make this evident (Warwick 2023). The Victorian government has declared their goal of being net zero by 2045, which includes the public health services acting under their remit (DEECA 2025). Despite this, our results indicate health promotion teams within organizations are failing to make clear the link between environmental and human health, and their initiatives are insufficient in shifting towards a net zero society.

We recognise public healthcare organizations with a dedicated health promotion team are often constrained by the priority areas and goals set by government. As a result, healthcare organizations are limited in their autonomy to focus on areas specific to their organization and local context. We see an opportunity for public health promotion teams to encourage their health service to improve end of trip facilities and increase active travel rates in their staff. Health promotion teams are likely to have an intricate understanding of their organizational barriers to issues such as active commuting and could be an invaluable asset to instigate cultural changes.

### The potential for end of trip facilities in healthcare policy

This analysis identified that no Victorian healthcare organization included in this study had a separate plan or policy regarding staff commuting. This is despite staff commuting being a major contributor to Scope 3 emissions and ultimately contributing to the overall emissions of the healthcare sector (Malik et al. 2018, Thanekar et al. 2024). The provision of end of trip facilities remains underdeveloped and under promoted in Victorian healthcare organizations, which has been shown to contribute to the low uptake of active travel options such as cycling and walking. Prior research suggests that investment in end of trip facilities is crucial in increasing the uptake of active travel options (Dill and Carr 2003, Heinen et al. 2013, Heinen and Buehler 2019, Ke 2024), with some health and transport practitioners going as far as to say that they are a ‘pre-condition for successful travel plan implementation’ (page 9) (Petrunoff et al. 2017). This is particularly the case for commuters, where facilities at work can be a deciding factor as to whether cycling is a feasible option (Heinen et al. 2013). Investment in end of trip facilities may be perceived as having a high up-front cost. However, this cost is inconsequential to the economic co-benefits that stem from improvements in health, the environment and society (Chapman et al. 2018, Ding et al. 2024, Ke 2024). Even traffic safety, a significant reason for low cycling uptake is shown to improve with greater rates of active travel (Ding et al. 2024).

### Strengths and limitations

We recognise this study contains limitations, which must be considered when interpreting the findings. This analysis did not include smaller rural healthcare organizations, which may impact on the transferability of findings to these contexts. Furthermore, our analysis included only publicly available information. While additional methods, such as organizational surveys or stakeholder interviews, may have provided further insights, these were beyond the scope and objectives of the current study. Future active travel research in the healthcare sector could build on this work by examining internal documents in greater depth, interviewing staff and organizational leaders. Finally, we took a focus on active travel, such as cycling and walking, as this achieves several environmental and human health co-benefits for individuals and organizations. However, other options are available to reduce employee commuting-related emissions, such as public transportation, working from home policies, and carpooling. These options also present limitations (e.g. provision of public transport services) but form an important part of a multifaceted solution and should be considered by policy makers and researchers.

This study also contains significant strengths. It is the only analysis of active travel policy within the healthcare sector in Australia that the authors are aware of at the time of writing. As such, it provides the sector with a baseline understanding of active travel policy to enable further investment and growth.

## CONCLUSION

This policy analysis of 21 public healthcare organizations in Victoria, Australia demonstrated a distinct lack of focus and accountability in increasing active travel rates within their staff. This is despite the majority of organizations signing on to the Global Green and Healthy Hospitals Initiative that incorporates patient and staff transportation as one of their 10 sustainability agenda items. While most organizations showcased an environmental sustainability policy, few were measurable and there remained little focus or direction to reducing the impact of their employee’s commuting. To shift organizational culture and support employee active travel, there needs to be a stronger emphasis on improving end-of-trip facilities and translating research into policies that reduce greenhouse gas emissions in healthcare organizations.

## Data Availability

Data made available upon request to the corresponding author.
